# Translation from the Gazette Des Hopitaux, Paris, Oct. 8, 1857

**Published:** 1858-03

**Authors:** 


*Translation from the Gazette des Hopitaux, Paris, Oct.* 8, 1857. *On the Difficulties in Diagnosis in certain Cases of Pneumonia. Clinical Lecture, Hotel Dieu Paris. By Professor* TROSSEAU. Transla ted by C. G. C.

Scarcely is one initiated into the mysteries of our art, when the diagnosis of a pneumonia is regarded as the easiest thing imaginable; and moreover this is che prevalent opinion in regard to diagnosis of the inflammation of the pulmonary parenchyma. A violent chill at first, then pain in the side; the first, second, and third days, bloody sputa, begining with the color of apricot marmelade, then that of prune juice, then that of rust, etc. Crepitant rale; blowing sound mingled with crepitant rale ; blowing sound alone;—bronchophony; in short, intense fever. Such are the leading symptoms of the disease, and he must be badly instructed who observes these morbid phenomena without recognizing them as he would a familiar acquanintance.

All this is very fine and very accurate as a mere book statement, but it is not always the same at the bedside. Listen now to the recital of sever-

---

also extirpated the supra-renal capsules, without having noticed the derangement of the central nervous system mentioned by Brown-Sequard. I should add, that I have seen two animals suffer from convulsons after extirpation of the capsules. One, a cat, died soon after, having slightly tetanic symptoms.

al cases which have recently occurred in my services here, which show how difficult a diagnosis may sometimes be.

A man aged fifty years entered the ward of St. Agnes on the fifth day of a Pneumonia. I came to his bed; I observed a subicteroid tint, a moderate difficulty in breathing, and I said there is a disease which resembles a peripnemonia. Why? I shall not tell you now, but in short, the expression of a peripneumonia is only like itself, and when one has seen only a hundred cases defile before him, he learns that such patients have a peculiar stamp.

I interrogated this man in a pneumonial point of veiw.

Have you had a chill? No.

Have you had pain in the side? No.

How did you take the fever? At first had some warmth; the heat augmented and I had a fever.

Do you feel pain in any part? When I take a full breath, I have more restraint on the left side than on the right, but I feel no pain.

Do you cough? Two or three times a day.

Do you expectorate? No.

But what do you spit up when you cough? I do not spit at all.

Nothing, in short, of sputa; and I put him in evry position without any manifestation of pain in the side.

Thus, then, there was no initial chill. Now this symptom is rarely absent in a pneumonia, and it is sufficient of itself in the largest number of cases to lead us to investigate for a pneumonia.

The pain in the side, the cough, the expectoration, were all absent.—We auscilted him and found nothing except the respiration slightly feeble at the summit of the left side. We went over again the stethoscopic examination; and at last on the side of the shoulder at the extreme end of the clavicle, in a circumscribed point not larger than a franc, piece, we perceived a little blowing and a very slight crepitant rale. In the axilla there was nothing heard, nor in the lesser scapular region, nor in any other part of the chest.

Behold now a pneumonia discovered, but you will understand that it was necessary to be very positive about it. My impressions were, however, just; for on that evening, even the blowing occupied all the subclavicular region;—the next day all the superior lobe, the next day all the inferior portion, and on the fourth day he died. His lungs are now before you, and they exhibit a pneumonia of the third stage.

This case shows you on one part that the diagnosis of pneumonia is not always easy, and on the other, that there really exists pneumonia which is essentially malign and suppurative; in effect, such a pneumonia does not follow in any degree the ordinary course, but enters in a trice the suppurative stage.

I need not add that the man was delirious during the two days of his life, which is always the case when death is inevitable from inflammation of the parenchyma of the lungs.

Lately I was called in consultation by one of my confréres for a young lady recently married, who had come from abroad to enjoy the pleasures of Paris for a season. She had been taken sick with severe chill but was much better the following day; on the succeeding one (or third day), she had another chill.

As the patient had come from a country where intermittent fever prevails regularly, her physician naturally thought her disease was a palustrian fever of the tertian type. The Sulphate of Quinia was administered and the paroxysms were arrested, but there remained a slight continued fever. Under these circumstances I saw her. She complained vaguely of a pain in the left side of the chest, but the most careful auscultation revealed, absolutly, no abnormal condition. The husband, the patient, and one waiting upon her, affirmed that there was neither cough nor expectoration. In veiw of the fever and a well marked sub-icteroid tint, we were led to fear a typhus affection; when on the eighth day of her attack she began to cough, and then could be heard a slight blowing at the base of the left lung. In the evening the blowing had increased, and in thirty-six hours after, it extended to the upper portion of the inferior lobe of the same lung, and a pneumonia was fully unmasked.—There was yet no expectoration, however, and scarcely five or six attacks of coughing in the course of the day and night.

Such cases as these you will often meet with in the course of your practice, therefore the duty of your being duly advised in regard to them.

You will remember that six days ago, I requested Professor Grisolle to come and give me the bnefit of experience in order to determine the question of a pneumonia or a pleurisy.

The case was that of a man fifty-three years of age in Saint Agnes Ward, who entered the Hospital with a dullness that occupied all the chest from top to bottom of the left side. The patient had a resonance of the voice resembling a bronchophony, but at the same time simulating egophony at several points. During several days while he remained here, there was no pneumonic expectoration; there were, though, some sputa, slightly green, but not tenacious. There had been no intial chill, but he was delirious during the first night he passed in Hotel Dieu. There was no pain in the side.

On ausculting the man with great care, we found readily this blowing from apex to base, and having in view the delirium and grave character of the symptoms, we decided on pneumonia; but the pain in the side and the chill being absent, we began again to be undecided.

On percussing, we found, as I have already said, an absolute dullness from top to bottom; it seemed more pleuritic than pneumonic. We investigated also for another sign, that is, the want of expansion of that side of the chest, (which never fails to exist in a well marked case of pleurisy,) remarking, however, the existance of a respiratory sound in all the anterior part, and even in the lateral region, phenomena which would not probably exist in a pleurisy, because, unless there had been pre-existent adhesion, the liquid would be level. *Cin. Lancet & Observer.*

